# Hold Breath: Autonomic Neural Regulation of Innate Immunity to Defend Against SARS-CoV-2 Infection

**DOI:** 10.3389/fmicb.2021.819638

**Published:** 2022-03-03

**Authors:** Changle Wang, Yan Zhao, Hongxiu Qiao, Zhiyun Gao, Jing Yang, Xia Chuai

**Affiliations:** ^1^Department of Pathogenic Biology, Hebei Medical University, Shijiazhuang, China; ^2^International Cooperation Laboratory of Stem Cell Research, Hebei Medical University, Shijiazhuang, China

**Keywords:** SARS-CoV-2, innate immunity, autonomic neural regulation, respiratory virus, respiratory inflammation

## Abstract

Severe acute respiratory syndrome coronavirus 2 (SARS-CoV-2) is a novel member of the genus of betacoronavirus, which caused a pandemic of coronavirus disease 2019 (COVID-19) worldwide. The innate immune system plays a critical role in eliminating the virus, which induces inflammatory cytokine and chemokine secretion, produces different interferons, and activates the adaptive immune system. Interactions between the autonomic nervous system and innate immunity release neurotransmitters or neuropeptides to balance the excess secretion of inflammatory cytokines, control the inflammation, and restore the host homeostasis. However, more neuro-immune mechanisms to defend against viral infection should be elucidated. Here, we mainly review and provide our understanding and viewpoint on the interaction between respiratory viral proteins and host cell receptors, innate immune responses to respiratory viral infection, and the autonomic neural regulation of the innate immune system to control respiratory viruses caused by lungs and airways inflammation.

## Introduction

At the end of 2019, an emerging severe acute respiratory infectious disease outbreak occurred in Wuhan, China (Hu et al., 2020). The causative agent that belonged to the genus Betacoronavirus of Coronaviridae family *via* complete genome sequence was identified and termed severe acute respiratory syndrome coronavirus 2 (SARS-CoV-2) by the World Health Organization. SARS-CoV-2 is an enveloped positive-sense single-stranded RNA (+ ssRNA) virus, which consists of nucleocapsid protein (N), membrane protein (M), envelope protein (E), spike protein (S), four structure proteins, 16 non-structural proteins (NSP1-NSP16), and nine putative accessory factors (Suryawanshi et al., 2021). The phylogenetic analysis reveals that, among all human infection-related beta coronaviruses, SARS-CoV-2 is in the same cluster with SARS-CoV that belongs to the subgenus Sarbecovirus. This virus is distant from Middle East respiratory syndrome coronavirus (MERS-CoV, Merbecovirus), human coronavirus (HCoV) HKU1, and OC43 (Embecovirus) in their phylogenetic relationship (Hu et al., 2020). The similarity of the whole genome sequences between SARS-CoV-2 and SARS-CoV is 79.6%. However, SARS-CoV-2 shares 96.2% and 93.3% identities of the genome sequences with bat coronavirus RaTG13 and RmYN02, so it was considered that SARS-CoV-2 might originate from bats (Hu et al., 2020).

SARS-CoV-2 was the seventh coronavirus to infect humans and caused a pandemic of coronavirus disease 2019 (COVID-19) worldwide. In addition to this newly emerging HCoV, four seasonal endemic coronaviruses (HcoV-NL63, HcoV-229E, HcoV-HKU1, and HcoV-OC43) and two epidemic coronaviruses (SARS-CoV and MERS-CoV) have been reported that infected human upper or lower respiratory tracts, which caused common cold and fatal pneumonia (Ovsyannikova et al., 2020). There are mild, critical, and severe types of clinical manifestations of COVID-19, and most of the confirmed patients showed the mild type (Wiersinga et al., 2020). The symptoms in the majority of the COVID-19 cases include fever, dry cough, fatigue, shortness of breath, and general malaise (Huang et al., 2020; Wiersinga et al., 2020). Furthermore, COVID-19–associated neurological diseases are also reported in clinical COVID-19 cases, such as encephalopathy, encephalitis, psychosis, and neurocognitive syndrome (Ellul et al., 2020). Many hospitalized patients exhibit headache, dizziness, loss of smell and taste, intracerebral hemorrhages, ischemic strokes, and even autonomic dysfunction, which were due to the direct effects of SARS-CoV-2 infection, para- or post-infectious immune-mediated neurological complications (Ellul et al., 2020; Iadecola et al., 2020).

The innate immune system is the first defense line protecting humans from being infected by microbial pathogens. It monitors pathogenic organism invasion and induces cytokine secretion to cause inflammation (Takeuchi and Akira, 2010). The intensity of inflammatory responses is very important for the host to control the pathogenic invaders’ infection because inadequate immune responses lead to host immunodeficiency, and excessive immune responses could damage the tissues or organs and even cause host death. The autonomic nervous system (ANS) has three nervous divisions, which include the sympathetic nervous system (SNS), the parasympathetic nervous system (PNS), and the enteric nervous system (ENS). The SNS and the PNS are composed of the nerve fibers projecting from the central nervous system (CNS) and sacral portion of the spinal cord, and the ENS is intrinsic because the neuron’s cell body is located inside the tissue. The ANS plays a pivotal role in regulating the innate immune responses through systemic, regional, and local pathways. The divisions of the ANS could be rapidly activated by the secreted inflammatory cytokines, chemokines, and other immune mediators and work together to balance immune dysregulation and eliminate pathogens (Rankin and Artis, 2018; Godinho-Silva et al., 2019). Previous studies elucidate that all these ANS division systems inhibit and control the inflammatory responses of several autoimmune diseases, bacterial and parasitic infections, *via* releasing noradrenaline or acetylcholine neurotransmitters and neuropeptides (Sternberg, 2006; Godinho-Silva et al., 2019). However, the studies and data on researching mechanisms of the interplay between the ANS and the innate immune system in defending against SARS-CoV-2 or other respiratory viral infections are limited. Based on the current research, we discuss and explain autonomic neural regulation of the innate immune responses to SARS-CoV-2 infection through adrenergic or cholinergic pathways and neuropeptides in this study.

## Respiratory Virus Binding Proteins and Host Cell Receptors

The interactions between respiratory virus proteins and host cell receptors are the preconditions of the viruses that infect and proliferate in human respiratory epithelial cells or immune cells. After binding to the receptors, the viruses enter into the cytoplasm of the host cells through fusion or endocytosis. The attachment proteins of SARS-CoV-2 and the selected previous human respiratory viruses include S protein (SARS-CoV-2, SARS-CoV, MERS-CoV, HcoV-NL63, 229E, OC43, HKU1), hemagglutinin (influenza A virus H1N1, H5N1, H7N9, measles virus), hemagglutinin-neuraminidase glycoprotein (parainfluenza virus, mumps virus), G glycoprotein (Hendra and Nipah virus, human metapneumovirus, respiratory syncytial virus), fiber protein (human adenovirus), E1 envelope glycoprotein (rubella virus), and VP1-VP2-VP3 complex protein (human rhinovirus), which could facilitate the respiratory viruses entry (Table 1).

**TABLE 1:** Summary of SARS-CoV-2 and selected previous human respiratory viruses.

Respiratory viruses	Types of nucleic acid	Viral attachment proteins	Binding receptors	Human adult cytokine storm	Diseases	References
Severe acute respiratory syndrome coronavirus 2	+ssRNA	Spike protein	Angiotensin-converting enzyme 2 (ACE2), tyrosine-protein kinase receptor UFO (AXL), neuropilin-1 (NRP1) and NRP2, asialoglycoprotein receptor 1 (ASGR1), kringle containing transmembrane protein 1 (KREMEN1) and CD147	+	Coronavirus disease 2019	Fung and Liu, 2019; Cantuti-Castelvetri et al., 2020; Daly et al., 2020; Hoffmann et al., 2020; Hu et al., 2020; Wang et al., 2020; Gu et al., 2021; Wang et al., 2021
Severe acute respiratory syndrome coronavirus			ACE2	+	Severe acute respiratory syndrome	
Middle East respiratory syndrome coronavirus			Dipeptidyl peptidase 4	+	Middle East respiratory syndrome	
Human coronavirus NL63			ACE2	–	Common cold	
Human coronavirus 229E			Aminopeptidase N	–		
Human coronavirus OC43			9-*O*-acetylated sialic acid	–		
Human coronavirus HKU1				–		
Influenza A virus H1N1	Segmented -ssRNA	Hemagglutinin	α-2,6-linked sialic acids (human), α-2,3-linked sialic acids (avian)	+	Influenza	Hartshorn, 2020
Influenza A virus H5N1						
Influenza A virus H7N9						
Parainfluenza virus	-ssRNA	Hemagglutinin- neuraminidase glycoprotein	Sialic acid	–	Common cold, bronchiolitis, pneumonia	Aguilar et al., 2016
				–		
Mumps virus				–	Mumps	
Measles virus		Hemagglutinin	Signaling lymphocyte activation molecule, CD46 and nectin-4	–	Measles	Aguilar et al., 2016
Hendra virus		G glycoprotein	Ephrin B2	–	Encephalitis, severe respiratory illness	Aguilar et al., 2016
Nipah virus			Ephrin B2, B3	–		
Human metapneumovirus			Heparan sulfate, Ca^2+^-dependent C-type lectin receptor DC-SIGN or L-SIGN or other glycosaminoglycans	–	Common cold, bronchiolitis	Aguilar et al., 2016; Gillespie et al., 2016
Respiratory syncytial virus			Nucleolin, intercellular adhesion molecule 1 (ICAM-1), CX3CR1 or heparan sulfate	–	Common cold, bronchiolitis, pneumonia	Johnson et al., 2015; Aguilar et al., 2016
Human adenovirus	dsDNA	Fiber protein	Coxsackie and Adenovirus Receptor, CD46, desmoglein-2, glycans GD1a or polysialic acid	+	Common cold, pharyngitis, pneumonia	Stasiak and Stehle, 2020
Rubella virus	+ssRNA	E1 envelope glycoprotein	Myelin oligodendrocyte glycoprotein	–	Rubella	Cong et al., 2011
Human rhinovirus	+ssRNA	VP1-VP2-VP3 complex protein	ICAM-1	–	Common cold	Norkin, 1995

On the other hand, angiotensin-converting enzyme 2 (ACE2) is reported as the binding receptor for SARS-CoV-2, SARS-CoV, and HcoV-NL63. In the case of SARS-CoV-2, the ACE2 receptor could be ligand to the receptor-binding domain (RBD) of the S protein, and the S1 and S2 ectodomain segments are demonstrated to function as a binding receptor and membrane fusion, respectively (Hoffmann et al., 2020; Hu et al., 2020). Dipeptidyl peptidase 4 (DPP4) is the receptor for MERS-CoV, aminopeptidase N for HcoV-229E, and 9-*O*-acetylated sialic acid for HcoV-OC43 and HKU1 (Fung and Liu, 2019). Influenza A viruses, parainfluenza virus, and mumps virus are demonstrated to utilize sialic acid as a receptor, whereas influenza A viruses bind to α-2,6-linked sialic acids on human epithelial cells and α-2,3-linked sialic acids on avian cells (Aguilar et al., 2016; Hartshorn, 2020). The remaining -ssRNA viruses, such as measles virus, Hendra and Nipah virus, human metapneumovirus, and the respiratory syncytial virus, can bind to Ephrin B2 and/or B3, signaling lymphocyte activation molecule, CD46, nectin-4, heparan sulfate, Ca^2+^-dependent C-type lectin receptor DC-SIGN or L-SIGN, nucleolin, intercellular adhesion molecule 1 (ICAM-1), CX3CR1, and other glycosaminoglycans (Table 1). In the case of human adenovirus, coxsackie and adenovirus receptor CD46, desmoglein-2, glycans GD1a, and polysialic acid are reported as binding receptors of this virus (Johnson et al., 2015; Gillespie et al., 2016; Stasiak and Stehle, 2020). Rubella virus and human rhinovirus can utilize myelin oligodendrocyte glycoprotein and ICAM-1 as receptors, respectively (Norkin, 1995; Cong et al., 2011). Remarkably, as we summarize in Table 1, one respiratory virus can bind to different types of receptors, and one host cell receptor can be utilized by several respiratory viruses (Table 1).

In addition, the tyrosine-protein kinase receptor UFO (AXL), asialoglycoprotein receptor 1 (ASGR1), kringle containing transmembrane protein 1 (KREMEN1), and CD147 are revealed to be ACE2-independent candidate receptors promoting SARS-CoV-2 infected human pulmonary, hepatic, and renal cell lines (Wang et al., 2020, 2021; Gu et al., 2021). AXL showed a different binding site from ACE2, which could interact with the S protein’s N-terminal domain (NTD), but not the RBD (Wang et al., 2021). ASGR1 and KREMEN1 were found that could interact with the RBD and NTD and the S2, RBD, and NTD, respectively (Gu et al., 2021). CD147-S protein is evidenced as a novel route for SARS-CoV-2 infection because CD147 mediates viral entering host cells by endocytosis (Wang et al., 2020).

SARS-CoV-2 showed extremely stronger infection ability because the type II transmembrane serine protease (TMPRSS2), proprotein convertase furin, and lysosome protease are reported to play a critical role in the cleavage of the S protein and promoting the virus entry (Hoffmann et al., 2020; Hu et al., 2020). After the S protein was cleaved into S1 and S2 by the furin protease, it generates a polybasic Arg-Arg-Ala-Arg carboxyl-terminal sequence on S1 that conforms to a C-end rule motif to bind to neuropilin-1 (NRP1) and NRP2 receptors, which significantly potentiates SARS-CoV-2 infectivity (Cantuti-Castelvetri et al., 2020; Daly et al., 2020). It is interesting that while ACE2, TMPRSS2, and NRP1 express in different types of neural cultures derived from human-induced pluripotent stem cells, SARS-CoV-2 could not efficiently replicate and spread in these cultures, but induced type III IFN and IL-8 secretion (Bauer et al., 2021). Furthermore, a recent study reported that non-muscle myosin heavy chain IIA (MYH9) could colocalize with S protein through interacting with the S2 subunit and S1-NTD by its C-terminal domain (PRA). Whereas endosomal or myosin inhibitors could effectively block SARS-CoV-2 entry of PRA-A549 cells, TMPRSS2 inhibitors could not, which indicates that MYH9 facilitates SARS-CoV-2 endocytosis and bypasses TMPRSS2 pathways (Chen et al., 2021).

## Innate Immunity Induces Proinflammatory Cytokine and Interferon Production to Virus Infection

The innate immune system is a rapid, non-specific, and conserved defense strategy for monitoring and defending invaded pathogens and activating acquired immune responses. The respiratory epithelial cells, innate immune cells, and soluble immune mediators are considered the front sentinel to form a barrier to defense against microbial infections. Toll-like receptors (TLRs, such as TLR2, TLR3, TLR7, and TLR8), and retinoic acid-inducible gene (RIG)-I-like receptors (RLRs, such as RIG-I, and melanoma differentiation-associated gene 5, MDA5) are reported to recognize viral ssRNA, double-stranded RNA (dsRNA), or DNA; subsequently recruit downstream adaptor proteins (such as mitochondrial antiviral signaling protein or myeloid differentiation primary response 88, MyD88); and activate interferon regulatory factor 3/7 or nuclear factor-κB (NF-κB) to induce type I/III interferons (IFN-I/III) or proinflammatory cytokines, such as tumor necrosis factor-alpha (TNF-α) and interleukin-6 (IL-6) secretion (Schildgen et al., 2011; Lee et al., 2020; Ovsyannikova et al., 2020; Vabret et al., 2020). Furthermore, the recruitment of MyD88 also could follow the MYD88-TRAF6 (TNF receptor-associated factor 6)-TAK1 (TGF-β-activated kinase 1)-p38-AP-1 (activator protein 1) pathway to induce IFN-I secretion (Fung and Liu, 2019). TLR and RLR mediated IFN-I production could induce the expression of IFN-stimulated genes by the infected and neighboring cells, which trigger in the host an antiviral state. In the case of the current emerging respiratory virus infection, SARS-CoV-2 infection inducing or antagonizing interferon responses remains elusive. SARS-CoV-2 triggers a particular signature that remarkably antagonized the IFN-I and IFN-III responses (Blanco-Melo et al., 2020), which elucidated association with the viral proteins of NSP1, NSP3, NSP15, ORF6, ORF7a/b, and ORF8 (Suryawanshi et al., 2021). However, SARS-CoV-2 infection has also been reported to induce IFN-I and III responses in COVID-19 patients, neural cultures, and human enteroids (Broggi et al., 2020; Bauer et al., 2021; Gilbert et al., 2021; Zhao et al., 2021).

**FIGURE 1:**
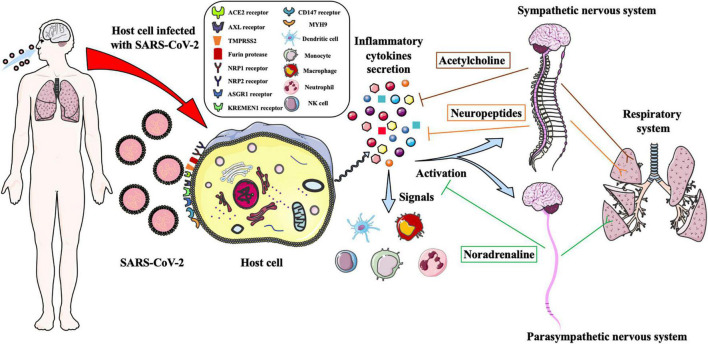
Schematic illustration of the autonomic nervous system regulates the innate immune response to defense against SARS-CoV-2 infection. SARS-CoV-2 infects target cell of human respiratory system through binding the ACE2, AXL, NPR1, NPR2, ASGR1, KREMEN1 and CD147 receptors, and could be facilitated cell entry *via* TMPRESS2, furin protease and MYH9. The infected cells are triggered to secrete massive proinflammatory cytokines, which could signal the immune cells to move to the viral infected sites and activate the sympathetic and parasympathetic nervous system to regulate the immune response. The noradrenaline and acetylcholine neurotransmitters and neuropeptides are produced to balance the cytokines secretion and bind to the adrenergic and cholinergic receptors to control the airways and lungs inflammation and regulate the functions of the respiratory system.

Generally, the proinflammatory cytokines can guide the innate immune cells to move to the virally infected sites and cause inflammation (Figure 1). Once massively produced, they work as a pathogenic factor to drive detrimental hyperinflammation and damage the host tissues and organs. SARS-CoV-2, SARS-CoV, MERS-CoV, influenza A viruses (H1N1, H5N1, H7N9), and human adenovirus are evidenced to trigger a strong dysregulated immune response and cause an adult inflammatory cytokine storm (Table 1; Hartshorn, 2020; Vabret et al., 2020; Li et al., 2021). SARS-CoV ORF3a and ORF8b proteins and unknown specific ligands of SARS-CoV-2 were elucidated that could stimulate nucleotide-binding oligomerization domain (NOD)-like receptors family pyrin domain-containing 3 (NLRP3) inflammasome activation; produce IL-1β, IL-6, and IL-18; and trigger murine and human macrophage pyroptosis (Lee et al., 2020; Rodrigues et al., 2021). On the other hand, SARS-CoV-2 infection was evidenced that enhanced the secretion of IL-1β, IL-2, IL-18, IL-6, IL-8, IL-7, granulocyte-colony stimulating factor (GCSF), and TNF-α (Blanco-Melo et al., 2020; Huang et al., 2020).

The cytokine storm was observed in severe cases of COVID-19, and high levels of serum L-6, IL-8, TNF-α were indicated that are associated with worse outcomes and shorter survival (Del Valle et al., 2020). Immune dysregulation is considered to play an important role in causing acute respiratory distress syndrome (ARDS) or respiratory failure in COVID-19 patients with macrophage activation syndrome or very low human leukocyte antigen D related expression (Giamarellos-Bourboulis et al., 2020). The mechanisms of these severe cases may be due to host immune hyperactivity, failure to balance the inflammatory responses, or inability to control the viral replication, and details should be elucidated further.

## The Autonomic Neural Regulation of Inflammation that was Caused by Immune Cells and Secreted Cytokines

The mechanisms of the interactions between the ANS and the innate immune system are very complex and are considered important immunoregulatory hubs. Neuro-immune communications are bidirectional because the innate immune cells and neurons can express neurotransmitter and cytokine receptors (Blake et al., 2019). Crosstalk between the ANS and the innate immunity through the SNS (adrenergic) and the PNS (cholinergic) play a critical role in regulating innate immune cell activation and proinflammatory cytokine production to control host inflammation. Whereas the SNS is reported to inhibit TLR-induced type I interferon responses, which supported virus replication (Collado-Hidalgo et al., 2006), vagal activity was hypothesized to upregulate Type I interferon response genes (Sloan and Cole, 2021). The SNS contains neuronal and hormonal components that can regulate the innate immune system through releasing noradrenaline and adrenaline from noradrenergic sympathetic nerve fibers and the medulla of adrenal glands, respectively (Sternberg, 2006). The binding of adrenaline to β-adrenergic receptors (β-AR) on intraparenchymal pulmonary mononuclear cells could suppress the levels of IL-1β, TNF-α, and IL-6 through activation of cyclic AMP (cAMP) and inhibition of NF-κB (Le Tulzo et al., 1997).

Furthermore, noradrenaline was immunosuppressive on dendritic cells (DCs), natural killer (NK) cells, macrophages, and monocytes by inhibiting IL-1, IL-6, IL-12, IFN-γ, and TNF proinflammatory cytokine secretion but could promote IL-10 anti-inflammatory cytokine production *in vitro*. Although noradrenaline is reported to increase the migration of monocytes, macrophages, and NK cells, it inhibits the migration of DCs and expression of chemokines CCL19 and CCL21 through upregulation of IL-10 (Sternberg, 2006). In a recent study, murine group 2 innate lymphoid cells (ILC2s) were demonstrated to express β2-AR, which was associated with reducing ILC2 immune responses and type 2 inflammation once treated with β_2_-AR agonist *in vivo* (Moriyama et al., 2018). Treatment of neutrophils with noradrenaline for 4 and 24 h can impair and reduce the neutrophil chemotaxis, activation, and phagocytosis (Godinho-Silva et al., 2019).

The PNS regulates the immune responses through afferent and efferent fibers of the vagus nerve to stimulate the inflammatory reflex (Tracey, 2009). Inflammatory products, such as IL-1, which binds to its receptor expressed on the paraganglia cells of the parasympathetic ganglia, then activate the vagus nerve’s afferent fibers. The activated afferent signals are transmitted to the nucleus tractus solitarius, subsequently activate efferent fibers of the vagus nerve to inhibit cytokine secretion *via* the cholinergic anti-inflammatory pathway, and provide negative feedback inhibition to control the inflammation (Wang et al., 2003). The principle parasympathetic neurotransmitter acetylcholine has nicotinic and muscarinic cholinergic receptor subtypes. The α7 nicotinic acetylcholine receptor (α7nAChR) is mainly expressed on macrophages, DCs, NK cells, and ILC2s, which play a cholinergic anti-inflammatory role in the stimulation of the vagus nerve and activation of the Jak2-STAT3 pathway. It can reduce the LPS-induced TNF, macrophage inflammatory protein-2, and IL-6 secretion in a dose-dependent way but not IL-10 (de Jonge et al., 2005; Zanetti et al., 2016). Vukelic et al. (2013) demonstrate that stimulation of α7nAChR on human polymorphonuclear neutrophils and blood mononuclear phagocytes reduced C5aR- and FcγR-triggered proinflammatory cytokines production and limited leukocyte recruitment and adhesion.

Neuropeptides can be released by neurons or nerve fibers of the ANS, such as neuropeptide Y (NPY), calcitonin gene-related peptide (CGRP), vasoactive intestinal peptide (VIP), and neuromedin U (NMU), which bind to G-protein-coupled receptors, and activate the secondary signaling pathways as signals to play the proinflammatory and anti-proinflammatory effects. NPY is a 36 amino-acid peptide synthesized and secreted from the sympathetic or enteric neurons, adrenal medulla cells, and immune cells, which could regulate and control the host inflammations through interactions with its receptors. Six subtypes of NPY receptors (Y1R-Y6R) widely expressed in mammalian innate immune cells, and the Y1R, Y2R, and Y5R were reported that mainly participated in the regulation of the host inflammations (Chandrasekharan et al., 2013). NPY is found that could play a stimulatory or inhibitory role in inactivation, differentiation, phagocytosis, migration, and proinflammatory cytokines secretion of macrophages, monocytes, DCs, NK cells, and neutrophils because the Y1R showed a bimodal effect on the mediation of these innate immune cells (Chandrasekharan et al., 2013; Chen et al., 2020). Furthermore, the Y1R mainly promotes the effect of NPY, whereas the Y2R and Y5R exert the suppression effect of the NPY (Chen et al., 2020).

CGRP is a 37 amino-acid neuropeptide that could downregulate host inflammation by inhibiting the activation and recruitment of macrophages, DCs, neutrophils, and ILC2s (Godinho-Silva et al., 2019). Nevertheless, it is demonstrated that human peripheral blood mononuclear cells could increase IL-1β, IL-6, and TNF-α secretion after CGRP administration *in vitro* (Sternberg, 2006). However, in a murine model *in vitro* and *in vivo*, CGRP inhibited the LPS-induced TNF-α production by macrophages but promoted LPS-induced IL-6 and IL-10 (Gomes et al., 2005).

VIP is a 28 amino-acid inhibitory neuropeptide, which acts as an immunoregulator to control inflammation *via* binding to its two G-protein-couples receptors. VIP could suppress the production of IL-6, TNF-α, IL-12, and chemokines by monocytes and stimulate the secretion of IL-10 by macrophages (Delgado et al., 1999; Chandrasekharan et al., 2013). VIP is also reported to inhibit the activity and activation of macrophages and NK cells but synergize with an optimal concentration of TNF-α to promote DCs maturation (Delneste et al., 1999; Sternberg, 2006). On the other hand, the VIP and NMU receptors are expressed in ILC2s, which the two types of neuropeptides could stimulate to produce IL-5 and IL-13 to trigger type 2 immune responses against anti-helminthic infection (Godinho-Silva et al., 2019).

Taken together, the ANS could regulate innate immune responses through adrenergic and cholinergic pathways and induce neuropeptide secretion, which mainly plays an anti-inflammatory role in inhibiting excessive proinflammatory mediator secretion and preventing inflammation deterioration. However, more detailed mechanisms of the neurotransmitters and neuropeptides in regulating innate immunity should be elucidated in future studies.

## The Autonomic Neural Regulation of Innate Immunity to Control Virus Infection–Caused Respiratory Inflammation

The SNS and PNS can regulate the functions of the respiratory system, which include airway and vascular smooth muscle tone, mucus gland secretion, bronchial flow and permeability, and inflammatory mediator release. Once barrier tissues of the respiratory system are under assault by microbial pathogens, such as bacterial, fungal, or viral infection, the triggered inflammation of airways and lungs could be regulated and inhibited through dense innervation of autonomic and sensory nerves (Blake et al., 2019). Neuro-immune interactions can maintain the integrity of the barrier cells and tissues and protect the respiratory system from being infected through antimicrobial mechanisms and clearance of the invaded pathogens.

Generally, respiratory viruses cause 90–95% of acute respiratory infections. These viruses could injure the human respiratory system and other tissues or organs outside the respiratory system, which caused human COVID-19, SARS, MERS, common cold, influenza, bronchiolitis, measles, mumps, encephalitis, pharyngitis, and rubella (Table 1). In a guinea pig model *in vivo*, parainfluenza virus infection was found to decrease M2 receptor mRNA expression and trigger M2 receptor of pulmonary smooth muscles and parasympathetic ganglia dysfunction and airway hyperreactivity but could be prevented by treatment with IL-1β and TNF-α antagonist anakinra (Rynko et al., 2014). Of note, the SNS could increase influenza A virus–induced proinflammatory cytokine and chemokine secretion and exacerbate the viral pathogenesis, which could be inhibited by treatment with α but not a β-adrenergic antagonist. Chemical sympathectomy by 6-hydroxydopamine (6-OHDA) decreased morbidity and mortality of influenza A virus–triggered pneumonia due to decreased inflammatory influx of monocytes, neutrophils, and NK cells (Grebe et al., 2010). In a recent study, severe influenza virus infection could increase the synthesis of NPY and Y1R, and deletion of *Npy* and *Y1r* genes could attenuate viral replication and proinflammatory cytokine secretion. NPY-Y1R could activate the suppressor of cytokine signaling 3 responsible for diminishing antiviral responses and promoting proinflammatory cytokine production, which enhanced the virus infection (Fujiwara et al., 2019).

In the case of SARS-CoV-2 infection, severe cases of COVID-19 have been reported that developed ARDS, acute lung injury, and even multiple organ failure, ultimately causing the patients’ death (Hu et al., 2020; Wiersinga et al., 2020). One possibility for causing ARDS was due to SARS-CoV-2 infection-induced endothelial dysfunction and immunothrombosis, in which activated neutrophils and monocytes aggregated with platelets and the coagulation cascade that caused microthrombi formation (Bonaventura et al., 2021). On the other hand, SARS-CoV-2 infection induced severe hyperinflammatory syndrome associated with dysregulated innate immune responses as aforementioned (Figure 1), which may result in severe tissue injury with vascular endothelial dysfunction and worsen pulmonary function micro thrombosis (Bonaventura et al., 2021). Of note, cigarette smoke is demonstrated to increase ACE2 expression (Smith et al., 2020), and it is hypothesized that ACE2 increase is induced by nicotine through α7nAChR (Russo et al., 2020). Interestingly, nicotine could activate the cholinergic anti-inflammatory pathway to attenuate the proinflammatory cytokine and chemokine production, whereas inhalation of nicotine may potentiate SARS-CoV-2 infectivity due to the upregulation of ACE2.

According to our understanding and viewpoint, the autonomic neural regulation of the innate immunity to control SARS-CoV-2 infection-induced inflammation is hypothesized that, after SARS-CoV-2 infects target cells of the human respiratory system, it leads to triggering much of the inflammatory cytokine secretion. The released cytokines bind to the receptors of the neurons to activate the SNS and PNS to secrete noradrenaline or acetylcholine neurotransmitters and neuropeptides, which bind to the receptors of the respiratory target cells to suppress excessive proinflammatory cytokine production and regulate physiological functions of the respiratory system to protect against viral infection (Figure 1). Furthermore, the released cytokines could also signal the immune cells to move to the viral infected sites to participate in innate immune responses (Figure 1). In mild cases of COVID-19, the ANS could inhibit overreaction of the innate immune responses, balance the levels of proinflammatory cytokines to prevent forming cytokine storms, and rapidly control the airway and lung inflammation through adrenergic and cholinergic pathways. Autonomic neural regulation of pro-inflammatory cytokine secretion and leukocyte recruitment *via* the released neurotransmitters and neuropeptides is also considered that could maintain vascular endothelial function homeostasis and prevent forming respiratory immunothrombosis. Because the neurotransmitters and neuropeptides are secreted moderately, the function of the respiratory system might be effectively regulated. Indeed, the ANS could not oversuppress the innate immune responses because excessive immunosuppression may lead to overwhelming viral infection.

In the critical or severe cases of COVID-19, many of the patients succumb to some basic diseases. The levels of the proinflammatory cytokines in the body may be imbalanced. Once infected with SARS-CoV-2, it may exacerbate the imbalance levels that form cytokine storms. The excessive secreted cytokines could recruit more innate immune cells to the virally infected sites to aggravate the hyperinflammation. Although neurotransmitter and neuropeptide production is considered to be increased, the autonomic neural regulation of the innate immune responses may be out of control. In addition, the secreted neurotransmitters and neuropeptides may continuously stimulate the target respiratory tissues and cells, worsen the functions of the airways and lungs, deteriorate the clinical manifestations and outcomes, and ultimately cause respiratory dysfunction and failure.

In summary, although the relevant research and experimental data that could directly elucidate the mechanisms of neuro-immune communications to defend against SARS-CoV-2 infection are still inadequate, we believe that the ANS can orchestrate to regulate the innate immune responses to eliminate infected SARS-CoV-2, restore and maintain innate immune homeostasis, and protect against hyperinflammation and cytokine storm to cause respiratory disorders.

## Conclusion

This study focused on ANS-mediated innate immune responses to defend against SARS-CoV-2 infection. We discussed the interactions of respiratory virus binding proteins and host cell receptors, the innate immunity induces proinflammatory cytokine and interferon production to antiviral infection, and the ANS regulates the antiviral infection role of the innate immune system to control respiratory inflammation.

SARS-CoV-2 has become a global threat, which caused more than 400 million people to be infected. Unfortunately, effective drugs or therapies of treatment for COVID-19 patients have not been found. Although nicotine is suggested to alleviate immune dysregulation and inhibit cytokine storm, inhalation of nicotine from tobacco could increase the risk of SARS-CoV-2 infection due to upregulation of ACE2. Two population-based clinical cohort studies revealed and analyzed that treatment of influenza, pneumonia, or ARDS patients with α1-adrenergic blockers could reduce 30-day mortality, risk of intensive care unit admission, mechanical ventilation, and death (Koenecke et al., 2021; Thomsen et al., 2021). In future studies, research and development of therapeutic strategies or drugs that can inhibit and counterbalance inflammatory cytokine secretion through adrenergic and cholinergic pathways are considered that may play an important role in helping us bring some enlightenment for treating COVID-19. According to our understanding, one possibility of the therapy strategies for treating the severe cases of COVID-19 might be stimulation of β-AR on the innate immune cells *via* binding noradrenaline neurotransmitters and blockage of α1-adrenergic receptors *via* its antagonists. The acetylcholine neurotransmitters or neuropeptides also could be employed for hyperinflammation and cytokine storm therapy strategies. Taken together, to better understand the autonomic neural regulation of SARS-CoV-2 infection triggered innate immune responses can provide a theoretical and experimental basis for clinical diagnosis and treatment of COVID-19.

## Author Contributions

CW, YZ, JY, and XC wrote and edited the manuscript. HQ and ZG searched the references. All authors contributed to the article and approved the submitted manuscript.

## Conflict of Interest

The authors declare that the research was conducted in the absence of any commercial or financial relationships that could be construed as a potential conflict of interest.

## Publisher’s Note

All claims expressed in this article are solely those of the authors and do not necessarily represent those of their affiliated organizations, or those of the publisher, the editors and the reviewers. Any product that may be evaluated in this article, or claim that may be made by its manufacturer, is not guaranteed or endorsed by the publisher.
